# Supplementary figures and images for: Introducing heat-not-burn tobacco improves hematocrit and cigarette smoking-related symptoms in patients with smokers’ polycythemia and polycythemia vera
Source: PLoS One. 2025 May 28;20(5):e0323437. doi: 10.1371/journal.pone.0323437 (PMC12118817; doi:10.1371/journal.pone.0323437)

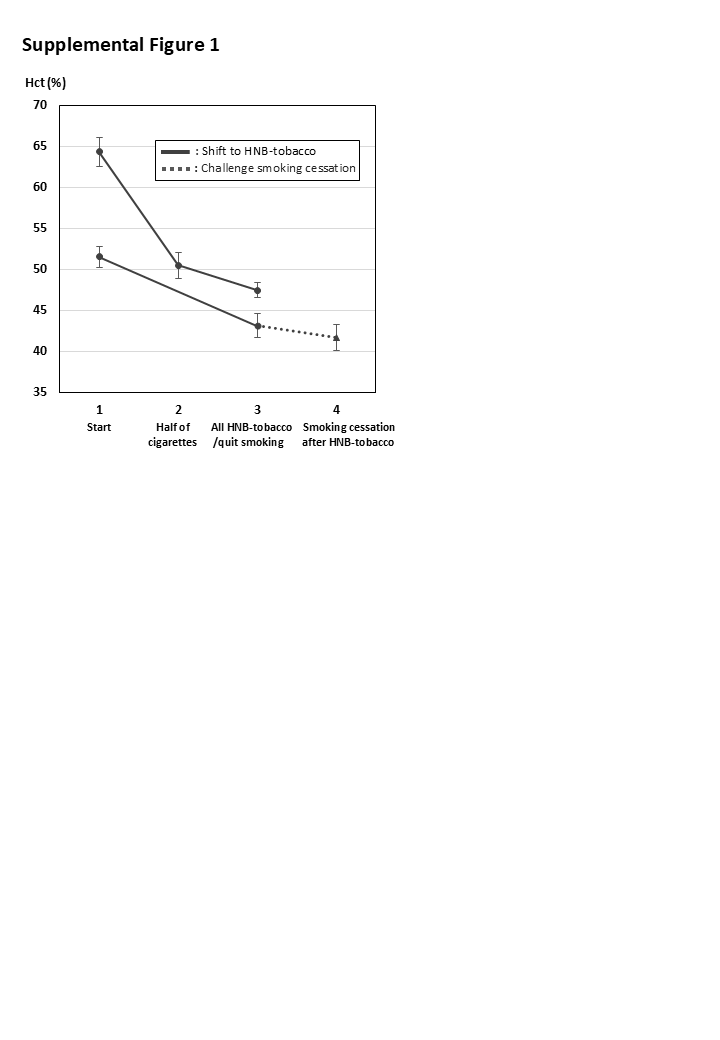

Supplement: S1 Fig — Solid line: changes in Hct related to a switch to heat-not-burn (HNB) tobacco; dotted line: changes in Hct after a switch to smoking cessation. *In patients with polycythemia vera, neither drug administration nor exsanguination volume changed during follow-up. (TIF) [file pone.0323437.s001.tif]
