# Supplementary material for: Introducing heat-not-burn tobacco improves hematocrit and cigarette smoking-related symptoms in patients with smokers’ polycythemia and polycythemia vera
Source: PLoS One. 2025 May 28;20(5):e0323437. doi: 10.1371/journal.pone.0323437 (PMC12118817; doi:10.1371/journal.pone.0323437)
Supplement: S1 Table — (DOCX) [file pone.0323437.s002.docx]

**Supplementary Table I.** Hematological data before a switch of habitual smoking

| No. | Diagnosis | Sex | Age | Cigarettes ( number /day ) | WBC (×10⁹/L) | RBC (×10^12^/L) | Hb (g/dl) | Hct (%) | PLT (×10⁹/L ) | D-dimer (×10⁹/L ) | BMI | Epo  (mIU/mL) |
| --- | --- | --- | --- | --- | --- | --- | --- | --- | --- | --- | --- | --- |
| 1 | **Smoker's polycythemia** | **Male** | **35** | **40-45** | **9.43±0.21** | **5.84±0.13** | **17.70±0.2** | **56.17±1.91** | **206.3±8.08** | **< 1.0±0.0** | **23.45** | **3.6** |
| 2 |  | **Male** | **72** | **25-30** | **5.80±0.35** | **5.62±0.12** | **19.63±0.35** | **56.90±0.35** | **114.7±15.04** | **N/A** | **20.4** | **4.4** |
| 3 |  | **Male** | **49** | **10** | **5.85±0.21** | **6.07±0.08** | **18.25±0.07** | **54.80±0.85** | **165.0±4.24** | **N/A** | **28.73** | **5.6** |
| 4 |  | **Male** | **62** | **20** | **6.63±0.70** | **4.47±0.12** | **16.93±0.32** | **51.00±1.59** | **847.3±4.93** | **< 1.0±0.0** | **22.07** | **3.2** |
| 5 |  | **Male** | **38** | **20** | **9.23±0.19** | **5.74±0.18** | **18.97±0.23** | **55.50±1.06** | **349.0±30.20** | **N/A** | **28.4** | **14.1** |
| 6 |  | **Male** | **50** | **30-35** | **10.02±0.99** | **5.49±0.12** | **16.70±0.47** | **51.40±0.44** | **383.3±33.41** | **< 1.0±0.0** | **27.54** | **5.3** |
| 7 |  | **Male** | **52** | **20** | **9.90±1.70** | **5.51±0.29** | **17.30±0.85** | **52.55±2.05** | **204.5±9.19** | **< 1.0±0.0** | **25.06** | **11.8** |
| 8 |  | **Female** | **55** | **20** | **7.83±0.45** | **5.48±0.16** | **17.87±0.31** | **53.90±0.46** | **320.3±37.50** | **< 1.0±0.0** | **20.95** | **5.1** |
| 9 |  | **Male** | **63** | **25-30** | **8.70±1.70** | **6.84±0.29** | **21.30±0.71** | **63.70±2.69** | **262.0±19.80** | **< 1.0±0.0** | **22.84** | **5.2** |
| 10 |  | **Male** | **64** | **20** | **5.73±0.35** | **5.73±0.03** | **18.50±0.10** | **54.33±0.21** | **362.3±12.22** | **N/A** | **22.62** | **4** |
| 11 |  | **Male** | **79** | **20** | **5.80±0.71** | **5.33±0.24** | **19.60±0.42** | **58.55±2.05** | **173.0±19.80** | **< 1.0±0.0** | **18.9** | **5.7** |
| 12 |  | **Male** | **57** | **20** | **9.15±1.77** | **5.82±0.08** | **17.80±0.56** | **54.73±1.62** | **332.0±3.54** | **< 1.0±0.0** | **21.09** | **37.2** |
| 13 |  | **Male** | **41** | **40** | **7.85±1.48** | **5.49±0.01** | **17.20±0.14** | **50.80±0.14** | **112.0±0.00** | **< 1.0±0.0** | **33.51** | **8.3** |
| 14 | **Polycythemia vera** | **Male** | **67** | **35-40** | **8.53±1.17** | **6.95±0.22** | **20.50±0.52** | **64.33±1.75** | **186.0±94.89** | **N/A** | **18.59** | **1.6** |
| 15 |  | **Male** | **68** | **10** | **6.40±0.28** | **5.71±0.09** | **16.8±0.14** | **51.50±1.27** | **362.5±60.10** | **< 1.0*** | **21.36** | **5.4** |

WBC; white blood cell count, RBC; red blood cell count, Hb; hemoglobin, HCT; hematocrit, RBC; platlet count, BMI; body mass index, Epo; Erythropoietin

N/A: Not applicable data ＊Only one point data
