# Supplementary material for: Introducing heat-not-burn tobacco improves hematocrit and cigarette smoking-related symptoms in patients with smokers’ polycythemia and polycythemia vera
Source: PLoS One. 2025 May 28;20(5):e0323437. doi: 10.1371/journal.pone.0323437 (PMC12118817; doi:10.1371/journal.pone.0323437)
Supplement: S2 Table — (DOCX) [file pone.0323437.s003.docx]

**Supplementary Table II.** Features of polycythemia vera patients after ≥2 month change to Heat-not-burn (HNB) tobacco

| No. | Patients group | Smoking cigarettes (number / day) | Hct (%) | | | WBC (×10³/μL) | | | symptom | |
| --- | --- | --- | --- | --- | --- | --- | --- | --- | --- | --- |
|  |  |  | **start** | **Half and half** | **After shift to**  **HNB tobacco** | **start** | **Half and half** | **After shift to**  **HNB tobacco** | **Start** | **After shift**  **After shift to**  **HNB tobacco** |
| 14 | **HNB tobacco** | **35-40** | **64.33±1.75** | **50.47±1.55** | **47.47±0.90** | **8.53±1.17** | **10.20±2.96** | **9.93±0.78** | **Heavy headedness**  **Dizziness･Fatigue**  **erythromelalgia** | **None** |
| 15 |  | **10** | **51.50±1.27** | **N/A** | **43.13±1.50** | **6.40±0.28** | **N/A** | **5.40±0.82** | **Heavy headedness erythromelalgia** | **None** |

HCT; hematocrit, WBC; white blood cell count N/A: Not applicable data

＊Half and half: Half and half number of smoking in HNB cigarettes and cigarettes
