# Supplementary material for: Introducing heat-not-burn tobacco improves hematocrit and cigarette smoking-related symptoms in patients with smokers’ polycythemia and polycythemia vera
Source: PLoS One. 2025 May 28;20(5):e0323437. doi: 10.1371/journal.pone.0323437 (PMC12118817; doi:10.1371/journal.pone.0323437)
Supplement: S1 File — (PDF) [file pone.0323437.s004.pdf]

Patients No.	Diagnosis	sex	age	height	weight	BMI	mutation	phlebotomy (within 3 months)	cigarettes (number/day)	WBC	RBC	Hb	Hct	PLT	D-dimer	Epo
1	Smoker's polycythemia	male	35	173	71.1	23.75	none	none	40-45	9200	596	17.9	56	215	< 1.0	3.6
										9600	570	17.5	53.8	205	1.1	
										9500	587	17.7	58.7	199	< 1.0	
2		male	72	165	60	20.4	none	none	25-30	6000	565	19.6	57.3	105	N/A	4.4
										6000	572	20	56.7	132	N/A	
										5400	548	19.3	56.7	107	N/A	
3		male	49	174	87	28.73	none	none	10	6000	613	18.2	55.4	162	N/A	5.6
										5700	601	18.3	54.2	168	N/A	
4		male	62	170.4	63.8	22.07	none	none	20	5900	454	17.2	52.2	853	< 1.0	3.2
										7300	433	16.6	49.2	844	< 1.0	
										6700	453	17	51.6	845	< 1.0	
5		male	38	178	90	28.4	none	none	20	7100	594	19.1	56.7	377	N/A	14.1
										10400	570	19.1	55.1	317	N/A	
										10200	559	18.7	54.7	353	N/A	
6		male	50	165	75	27.54	none	none	30-35	9400	545	16.5	51.2	355	< 1.0	5.3
										11300	540	16.3	51.1	375	N/A	
										9900	562	17.2	51.9	420	< 1.0	
7		male	52	169.5	72	25.06	none	none	20	11100	571	17.9	54	211	< 1.0	11.8
										8700	530	16.7	51.1	198	< 1.0	
8		female	55	163.6	56	20.95	none	none	20	7800	531	17.6	53.5	320	< 1.0	5.1
										7400	550	17.8	54.4	283	< 1.0	
										8300	563	18.2	53.8	358	< 1.0	
9		male	63	170	66	22.84	none	none	25-30	9900	700	21.8	65.6	276	< 1.0	5.2
										7500	668	20.8	61.8	248	< 1.0	
10		male	64	165.3	61.6	22.62	none	none	20	5400	570	18.4	54.1	365	N/A	4
										6100	574	18.5	54.5	373	N/A	
										5700	575	18.6	54.4	349	N/A	
11		male	79	172	56	18.9	none	none	20	6300	567	19.9	60	173	1.3	5.7
										5300	533	19.3	57.1	201	< 1.0	
12		male	57	157	52	21.09	none	none	20	7900	588	17.2	53.8	340	< 1.0	37.2
										10400	573	17.9	53.8	345	< 1.0	
										9100	586	18.3	56.6	311	N/A	
13		male	41	171	98	33.51	none	none	40	8900	550	17.3	50.7	112	< 1.0	8.3
										6800	548	17.1	50.9	112	< 1.0	
14	Polycythemia vera	male	67	172	55	18.59	JAK2V617F	none	35-40	9800	707	20.8	64.8	294	N/A	1.6
										7500	709	20.8	65.8	148	N/A	
										8300	670	19.9	62.4	116	N/A	
15		male	68	169	61	21.36	JAK2V617F	none	10	6200	577	16.9	52.4	405	< 1.0	5.4
										6600	564	16.7	50.6	320	N/A	
