# Supplementary material for: Introducing heat-not-burn tobacco improves hematocrit and cigarette smoking-related symptoms in patients with smokers’ polycythemia and polycythemia vera
Source: PLoS One. 2025 May 28;20(5):e0323437. doi: 10.1371/journal.pone.0323437 (PMC12118817; doi:10.1371/journal.pone.0323437)
Supplement: S2 File — (PDF) [file pone.0323437.s005.pdf]

No.	Patients group	Hct (%)			WBC ($\times 10^3/\mu$L)		
		start	Harf of tabaco amount	After shift e-cigarette or quit smoking	start	Harf of tabaco amount	After shift e-cigarette or quit smoking
1	Smoker's polycythemia	56	53.3	49.7	9200	7700	7800
		53.8	51.5	49.9	9600	7700	7900
		58.7	49.9	49.3	9500	7100	7600
2		57.3	53.3	46.5	6000	5600	5200
		56.7	52.8	44.2	6000	5300	5500
		56.7	51.2	47.4	5400	5400	5000
3		55.4	N/A	48	6000	N/A	5200
		54.2	N/A	49.8	5700	N/A	5900
		N/A	N/A	47.9	N/A	N/A	5800
4		52.2	47.2	40.6	5900	8900	8900
		49.2	43.9	40.5	7300	12800	9500
		51.6	N/A	41.5	6700	N/A	8600
5		56.7	N/A	50.8	7100	N/A	10400
		55.1	N/A	51.3	10400	N/A	8500
		54.7	N/A	N/A	10200	N/A	N/A
6		51.2	49.9	46.6	9400	11300	9700
		51.1	49.5	49.5	11300	10500	6900
		51.9	N/A	48.8	9900	N/A	8600
7		54	N/A	52.1	11100	N/A	8600
		51.1	N/A	49.8	8700	N/A	11000
8		53.5	N/A	45.2	7800	N/A	6700
		54.4	N/A	44.2	7400	N/A	7200
		53.8	N/A	N/A	8300	N/A	N/A
9		65.6	55.6	49.1	9900	10000	7900
		61.8	57	47.3	7500	9700	9800
10		54.1	N/A	42.7	5400	N/A	8500
		54.5	N/A	41.6	6100	N/A	7800
		54.4	N/A	40.4	5700	N/A	14000
11		60	N/A	48.3	6300	N/A	4700
		57.1	N/A	48	5300	N/A	6600
12		53.8	50.1	N/A	7900	11400	N/A
		53.8	51.8	N/A	10400	8800	N/A
		56.6	48.1	N/A	9100	7600	N/A
13		50.7	N/A	47.6	8900	N/A	8100
		50.9	N/A	N/A	6800	N/A	N/A
14	Polycythemia vera	64.8	51.1	47.4	9800	9900	9300
		65.8	51.6	46.6	7500	7400	9700
		62.4	48.7	48.4	8300	13300	10800
15		52.4	N/A	44.8	6200	N/A	4800
		50.6	N/A	41.9	6600	N/A	6000
