# Supplementary material for: Introducing heat-not-burn tobacco improves hematocrit and cigarette smoking-related symptoms in patients with smokers’ polycythemia and polycythemia vera
Source: PLoS One. 2025 May 28;20(5):e0323437. doi: 10.1371/journal.pone.0323437 (PMC12118817; doi:10.1371/journal.pone.0323437)
Supplement: Attachment — Submitted filename: Response to Reviewers Plos_one_smokers_porycythemia.docx [file pone.0323437.s009.docx]

**Response to Reviewer: 1**

We wish to express our appreciation to the reviewer for his or her insightful comments, which have helped us improve the paper.

- Did smokers polycethemia patient - besides the heat-not-burn (HNB) tobacco- undergo therapeutic venesection (phlebotomy)?? ((- as this procedure widely and commonly used to decrease hematocrit in smokers polycethemia to reduce hyperviscosity and consequently reduce incidence of thrombosis))

Response: Thank you for pointing this out. Two patients had undergone phlebotomy (polycythemia vera; n=1, smokers’ polycythemia; n=1), but they had stopped phlebotomy more than three months before the start of the clinical trial. Phlebotomy was not resumed during the study period.

The following explanation has been added to the "Treatment administration and assessment of outcomes" in the Methods section.

→

**“Two patients had undergone phlebotomy but had discontinued phlebotomy more than 3 months before entering this study, and there was no resumption of phlebotomy during the observation period.”**

- In method, some details should be mention like the study area (high attitude….), medical history of patients (obesity, cardiac disease, obstructive sleep apnea), Also definition and details regarding (HNB) tobacco using.

Response: Thank you for pointing this out. The following explanation has been added to the "Diagnosis" of the Methods section. We described the fact that we ruled out any medical history that could be a cause of secondary polycythemia other than smoking and added the method for diagnosing smoking polycythemia. In addition, information about altitude has been added to the "Patient" section of the results.

→

***“*Diagnosis**

**The patient differentiation process is shown in Figure 1. In smokers with polycythemia, causes of secondary polycythemia other than smoking were excluded: obesity (BMI ≥ 35), diabetes mellitus, psychological stress, sleep apnea syndrome, hyperthyroidism (hypertensive patients only). In addition, it was confirmed that erythropoietin was normal or low, and *BCR-ABL* gene fusion and *JAK2* mutations (V617F and exon 12 mutation) were negative. Furthermore, patients who were negative for *JAK2* mutations, whose bone marrow biopsy did not show hypercellularity with trilineage growth, were diagnosed as having smokers’ polycythemia.”**

**Results**

**Patients**

“Of the 40 patients with polycythemia who were smokers, the cause of polycythemia was investigated and differentiated in 26 patients. **They lived between 20 and 70 m above sea level.**”

- In method, patient, line -6 -((.. From August 1, 2009 to December 31, 2021, smokers who met the WHO 2016 diagnostic criteria for hematocrit (Hct) and Hb of polycythemia vera (Hct >49% and/or Hb 16.5 g/dL in males; Hct >48% and/or Hb 16.0 g/dL in females) who had failed a challenge of smoking cessation were recruited The patient differentiation process is shown in Figure 1)). Please recheck sentence as according figure 1 a fifteen where recruited, They quit smoking, and switch to HNB tobacco (NOT fail a challenge of smoking cessation.

Thank you for pointing this out. Figure 1 and the text have been modified.

→

**“From August 1, 2009, to December 31, 2021, we recruited smokers who met the WHO 2016 hematocrit (Hct) and Hb diagnostic criteria [10] for polycythemia vera (Hct >49% and/or Hb 16.5 g/dL in males; Hct >48% and/or Hb 16.0 g/dL in females) and who had a history of failing a challenge of smoking cessation in the past.”**

- The study was from August 1, 2009 to December 31, 2021, that’s mean the study period was 11 years. How many patients of smokers polycethemia included in that period ? Can you compare hematocrit between smokers with polycythemia who failed the smoking cessation challenge and others who chose to quit smoking, or switch to tobacco HNB. Clarifying whether smokers with polycythemia who fail a smoking cessation challenge receive treatment or therapeutic phlebotomy. I believe that this clarification is needed in the study because the study period was long and the sample enrolled was small.

Response:

Thank you very much for your excellent suggestion.

We identified 40 smokers with polycythemia, and 26 patients were examined for other causes of polycythemia. Seven of the 26 patients were excluded owing to obesity (n=2), diabetes mellitus (n=4), or mental stress at work (n=1). Two of the 19 patients were JAK2V617F positive and were diagnosed with polycythemia vera. Seventeen were diagnosed with smokers’ polycythemia, but four chose to continue smoking. Thirteen patients with smokers' polycythemia and two patients with polycythemia vera quit smoking or switched to HNB tobacco. We have added these explanations to the “Patients” section in the Results.

No patients resumed smoking cigarettes after switching to HNB tobacco. However, two of the four patients who chose to quit smoking resumed their smoking habits. We have added this information to the “Long-term changes in the smoking habit” section of the Results.

→

**Results**

**Patients**

**“Of the 40 patients with polycythemia who were smokers, the cause of polycythemia was investigated and differentiated in 26 patients. They lived between 20 and 70 m above sea level. Seven patients were excluded owing to obesity (BMI>35; n=2), diabetes mellitus (n=4), or mental stress at work (n=1). Two of the 19 patients were JAK2V617F positive and were diagnosed with polycythemia vera. Seventeen patients were diagnosed with smokers’ polycythemia, but four did not participate in the study because they continued smoking (Figure 1). Therefore, 13 patients with smokers’ polycythemia and two patients with JAK2V617F-positive polycythemia vera were enrolled in this study (Figure 1).”**

＊Reasons why the number of registered patients was small compared to the registration period

1. Only patients with a history of failed smoking cessation challenges in the past were recruited.

2. There was a lack of tests to determine the differential diagnosis, which limited the number of eligible patients.

3. Physicians other than hematologists were not yet accurately aware of the complications (eg, thrombosis) risks of secondary polycythemia, and referrals to the hematology department were rare.

4. Owing to institutional ethical standards, only patients referred to the Department of Hematology were allowed to participate in the study.

It was not possible to include in the main text the follow-up of four smoking polycythemia patients who were not enrolled in the study (continued to smoke). However, for patients whose smoking amount returned to pre-study levels, Hct level returned to the same level at the enlloled of this study. We have added this fact to the end part of "Long-term changes in smoking habits" section.

→

**Long-term changes in the smoking habit**

**“Patients who returned cigarette consumption to before smoking cessation, their Hct level returned to the same level at the enrollment in this study (data not shown).”**

As reference, we write down the changes of Hct and WBC in four smokers’ polycythemia patients who continued to smoking.

→

“No change was observed in the WBC or Hct of the four patients who continued smoking (WBC, 9150±1277/µL vs. 10650±1546/µL, p=0.400; Hct, 53.8±3.26% vs. 55.75±2.36, p=0.629).”

**Response to Reviewer: 2**

We wish to express our appreciation to the reviewer for his or her insightful comments, which have helped us improve the paper.

-The ideas behind the manuscript are clear, however, some of the concepts and sentence structures may have been lost in translation.

Response:

Thank you for pointing this out. We reviewed the text and added or modified missing explanations. Our manuscript has undergone professional English editing to ensure correct grammar and a native English tone.

**Abstract**

- Objectives as a first heading should be changed to Introduction instead.
- Remove “on”.
- Methods section in abstract requires refinement.

Response:

Thank you for pointing this out. We deleted the objectives from the abstract and included this information in the introduction. In addition, we removed “on.”

Following PLOS One's style also solved the problem in the Methods section.

**Introduction**

- A reference at the end of the sentence would suffice and prevent the flow of the sentence from being interrupted.
- Also consider HIF as a mechanism for absolute erythrocytosis (esp in patients with COPD)
  - However, the authors should mention and refer to reputable sources that EPO is low in smokers, in contrast to other causes of secondary polycythaemia where an elevated serum EPO level is to be expected.

Response:

Thank you for pointing this out. We have added a statement that smokers tend to have polycythemia even with low EPO levels, and we added one more article to the references.

Grammar and sentence structures should be checked to ensure that the correct words and terms match the meaning/intention of the sentences. This seems to have been lost in the translation of the manuscript.

Response:

Thank you for pointing this out. The references have been changed to only be listed at the end of the text. However, some references were left in the middle of the text to make it easier for readers to search.

**Methods:**

- Please write genes and fusion genes such as “*BCR::ABL1*” in Italics instead of “BCR-ABL”

Response: Thank you for pointing this out. I have corrected it as you pointed out.

- FISH sensitivity to detect *BCR::ABL1* is very low; why was PCR not used and were there any clinicopathological features of CML in any of these patients?

Response:

To differentiate polycythemia vera, it is necessary to confirm that *BCR-ABL* is negative (WHO 2017 diagnostic criteria). Therefore, *BCR-ABL* was measured even in the absence of typical clinicopathological features of CML.

The accuracy of FISH and the reasons for conducting it are as follows.

1. When we contacted SRL, we found that FISH had the same detection level as 10*2 copies of PCR.

2. The FISH diagnostic testing method is recommended, like PCR, in the NCCN (National Comprehensive Cancer Network) guidelines and the Japanese Guidelines.

3. There are very few facilities that can measure micro *BCR-ABL* using the PCR method. Japan Health Insurance requires the PCR method to measure major BCR-ABL and minor BCR-ABL in different months. In addition, measuring micro *BCR-ABL* requires more time.

Since the purpose of this study is actual medical treatment, we proposed a method that is in line with these guidelines and actual clinical practice. We also believe that simpler methods are more versatile worldwide.

- Define “SRL” before using it as an abbreviation.

Response: Thank you for pointing this out. I checked with the company, but SRL is the official name (not an acronym or an abbreviation). We have changed the name to SRL, Inc., to avoid misunderstanding.

- Provide more detail in the “Gene analysis” section.
  - Was a positive/mutant (heterozygous/homozygous), a negative/wild-type and a reagent blank control used during the *JAK2* mutation test?
  - Please state whether the PCR products (end-point analysis) were read on gel cards.

Response: I checked with the company. AS (Allele Specific) PCR uses the same reverse primer and two different forward primers for the normal allele and the mutant allele to compete in the PCR reaction to semi-quantitatively measure the *JAK2* mutation, which is a single nucleotide mutation. Therefore, it is possible to quantify Allele burden. Real-time PCR uses Qiagen's rotor G, so no gel card is used.

- Define what exactly is meant by ‘heat-not-burn’ (HNB) tobacco in the method section under “Treatment administration and assessment of outcome”.

Response: Thank you for pointing this out. Added that HNB tobacco used Ploom S, Ploom X, and IQOS ILUMA.

Was the serum EPO level recorded, if not, why not?

Response: EPO levels are listed in Supplementary Table 1. A comparison between the HNB tobacco group and the non-smoking group is also listed in Table 1. Because this study focused on changes in Hct and clinical symptoms, EPO was measured only at the first visit. Additionally, frequent EPO measurements were not performed in this study because they are not permitted by Japanese guidelines or the insurance system.

- Which programs/software were used for the statistical analysis and graphical representation of the data?

Response: We used R soft. We added this information to the Methods section.

- How was the sputum volume objectively measured or is this also a subjective assumption?

Response: It was confirmed that the number of times spittoon per day was 0. Therefore, there is no doubt that their sputum completely disappeared after switching to HNB cigarettes, but we did not measure the amount.

- Hct is the only objective measure of improvement.
  - Was % blood oxygen saturation assessed for each of the patients at baseline and follow-up?

Response: Blood oxygen saturation was not measured. This is because oxygen saturation has not been shown to be involved in thrombosis or mortality in secondary polycythemia, including smoking polycythemia. The guidelines do not include SPO2 as an observation item, and Hct is the main indicator for treatment. However, with regard to respiratory status, all patients were grade I on the Fletcher-Hugh-Jones classification and grade 0 on the MRC (Medical Research Council) dyspnea scale.

・ The authors mention a mechanism of reduced plasma cell volume with cigarette smoking. Was systemic blood pressure affected by the switch to HNB or smoking cessation?

Response; Plasma cells differentiated from B lymphocytes are not described. Plasma volume is not measured or calculated because it is difficult to measure directly, and it is easily affected by eating, drinking, and other physical conditions. Blood pressure was not measured because the body adjusts it to some extent even if there is a change in circulating plasma volume. However, systolic blood pressure was measured at every consultation, and it was 100 before switching vs. after 200 switching (p=0.81).

- Were baseline lung function tests performed?

Response; Thank you for pointing this out. We did not perform lung function tests. However, all patients were grade I on the Fletcher-Hugh-Jones classification and grade 0 on the MRC (Medical Research Council) dyspnea scale. In addition, no patients were diagnosed with COPD on chest X-ray. We have added this information to the Patients section of the Methods.

- Is there a follow-up planned for incidence of thromboembolism in these patients?

Response: We are in the process of increasing the number of participating hospitals (currently under review by the Ethics Committee), and we plan to add not only thrombosis but also larger-scale and more detailed analyses (COHb measurements, secondary cancer rates, etc.).

**Results**

Table 1 – Total number is 12 but HNB is 8 and smoking cessation is 5 which equals 13. Was the one patient that had an unsuccessful smoking cessation, excluded from the data? I agree with the exclusion, but the authors need to state this exclusion and change the values in the table.

Response: Thank you for pointing this out. The total number is 13. The total number in Table1 was incorrect. We corrected it.

**Discussion**

“One possible mechanism is that absolute erythrocytosis which activates thrombin21 and increases platelet P secretin expression,22 which in turn increases the risk of thrombosis.” Did the authors mean “P selectin”?

Response: Thank you for pointing this out. We corrected it as P selectin.

“As the risk of secondary carcinogenesis related to cigarettes, cigarette smoking increases the incidence of polycythemia vera,26,27 as well as 16 types of cancer: lung, upper aerodigestive tract (oral cavity, nasal cavity, nasal sinus, pharynx, larynx), esophagus, stomach, pancreas, liver, kidney, lower urinary tract (renal pelvis and urinary bladder), and uterine cervix cancers, myelogenous leukemia, and myeloproliferative neoplasms. 28” This sentence seems incomplete.

Response: Thank you for pointing this out. The text has been modified.

→

Regarding the risk of secondary carcinogenesis related to cigarettes, cigarette smoking increases the incidence of 17 types of cancer: lung, upper aerodigestive tract (oral cavity, nasal cavity, nasal sinus, pharynx, larynx), esophagus, stomach, pancreas, liver, kidney, lower urinary tract (renal pelvis and urinary bladder), and uterine cervix cancers, myelogenous leukemia, and myeloproliferative neoplasms (including polycythemia vera). [26, 27, 28]

Any number less than 10 can be written out in full, unless it is a measured value. E.g “…observed between the 2 groups before the change in smoking habits” should be written as two instead of 2.

Response: Thank you for pointing this out. The text has been modified.

A comment in the discussion section and in the summary should make it clear that there is no evidence that tobacco products that are not burnt are less harmful than cigarettes and that these products should be used with caution. These products should also not be endorsed by health professionals.

The methodology and results of this study are sound and contain very interesting findings, but the text needs a grammar/sentence reconstruction/spell check revision before this article is considered for publication.
