# Supplementary material for: Introducing heat-not-burn tobacco improves hematocrit and cigarette smoking-related symptoms in patients with smokers’ polycythemia and polycythemia vera
Source: PLoS One. 2025 May 28;20(5):e0323437. doi: 10.1371/journal.pone.0323437 (PMC12118817; doi:10.1371/journal.pone.0323437)
Supplement: Attachment — Submitted filename: Response to Reviewers_comments_for_revised.docx [file pone.0323437.s010.docx]

We wish to express our appreciation to the reviewers again for his or her comments, which have helped us improve the paper.

Could the authors of this study please provide a conflict-of-interest statement.

→In addition to the COI, we have also added Author contributions and a Data Availability Statement.

HUGO Gene Nomenclature Committee (HGNC) recommendations for the designation of gene fusions, recommends writing a fusion gene as ***BCR::ABL1****.*

- Bruford, E.A., Antonescu, C.R., Carroll, A.J. et al. HUGO Gene Nomenclature Committee (HGNC) recommendations for the designation of gene fusions. Leukemia 35, 3040–3043 (2021). <https://doi.org/10.1038/s41375-021-01436-6>
- Also see Nomenclature under the Submission guidelines for PLOS ONE (<https://journals.plos.org/plosone/s/submission-guidelines>)

The term ‘variant’ should be used instead of ‘mutation’ throughout the manuscript.

Subject to the requirements of PLOS ONE, it should be sufficient to place a reference at the end of the sentence and to improve the "flow" of the sentence.

→I have corrected it as you pointed out.

**Abstract**

Line 35: write 2 as “two” instead.

→I have corrected it as you pointed out.

**Introduction**

Line 52: mechanism or plural mechanisms? Change the grammar accordingly.

→It is plural because it refers to the mechanisms in lines 48-52.

Line 58: Treatment for smokers’ polycythaemia?

→ Generally, patients with smoker’s polycythemia are not given medical treatment. However, guidelines indicate phlebotomy if the Hct is >55% and/or the patient is symptomatic. In this study, as described in the "Treatment administration and assessment of outcomes" section of the Methods, two patients had undergone phlebotomy, one of whom had been diagnosed with smoker’s polycythemia. However, as described in “Treatment administration and assessment of outcomes” section of the manuscript, all patients had discontinued phlebotomy >3 months before entering this study. Therefore, patients with smoker’s polycythemia were not receiving treatment at study initiation.

Line 59: suggest using “detection or analysis or testing” rather than “measurement”

→I have corrected it as you pointed out.

Line 60-61: Do the authors mean that the distinction/differentiation between a diagnosis of PV and secondary polycythaemia has become more accurate?

→Thanks, you are right.

Line 59-63: This needs clarification. Do the authors mean that because we are now able to better distinguish between smokers’ (i.e secondary) polycythaemia and polycythaemia vera by using JAK2 variant analysis, that the results of the study in reference 10, in which JAK2 testing was performed, is more accurate than the 1993 report (case-control study), in determining which condition is more associated with thrombosis?

If this is the case, I suggest that the authors instead mention that the fact that JAK2 variants were not tested for is a limitation of the 1993 report and not the reason why the results of reference 10 suggest an equivalent thrombosis rate. Example: “In contrast to these earlier reports, where PV was not confirmed by testing for the JAK2 variant, a more recent study has shown that secondary polycythaemia and polycythaemia vera have comparable rates of thromboembolic events. This suggests that secondary polycythaemia also requires intervention to reduce the risk of thrombosis in this cohort.”

→I have corrected the sentence as per your advice.

[The authors should also consider this article (Nadeem O, Gui J, Ornstein DL. Prevalence of venous thromboembolism in patients with secondary polycythemia. Clin Appl Thromb Hemost. 2013;19(4):363-366. doi:10.1177/1076029612460425) which states that “…secondary polycythemia alone may not be a significant risk factor for VTE but that VTE risk in this population may be related to known risk factors such as obesity.”]

→I am aware of this study. However, it focuses only on venous thrombosis. As described in references 10, 26, and 27, both secondary polycythemia and PV have higher risks of thrombosis in the arteries than in the veins. In addition, the study you mentioned included patients with severe obesity (Max BMI of 59.5 kg/m^2^). In contrast, the present study excluded participants with severe obesity (the maximum BMI in this study was 33.51 kg/m^2^). Generally, a BMI >30 kg/m^2^ is associated with an increased risk of venous thrombosis due to obesity. However, all but one patient had a BMI <30 kg/m^2^. Therefore, I believe that the study you mentioned is not relevant to the present study and should not be cited.

Line 68: I suggest: “…increased the need for consultation with a hematologist.”

→I have corrected the sentence as per your advice.

Line 75-77: The authors are making a bold statement about HNB tobacco as a ‘therapeutic aid’. Please rephrase this statement.

→The text has been corrected to read as follows.

“we report that switching to HNB tobacco improved smokers’ polycythemia and subjective symptoms with smoking in smokers. ”

**Method**

Line 112-113: Add the abbreviation after polymerase chain reaction (PCR), which is used later in the paragraph, and move the AS abbreviation to allele-specific instead of putting it after 'polymerase'.

→Thank you for your advice. I Corrected fixed it.

Please define FISH in the manuscript before using the abbreviation and provide sufficient methodological details in the manuscript for “FISH at SRL, Inc” and the PCR used for JAK2 testing.

→ “FISH” has been corrected to "fluorescence in-situ hybridization." Regarding the PCR, any information beyond that reflected in the response to the previous peer reviewer is confidential and cannot be provided by SRL, Inc. Therefore, I apologize that I cannot provide additional details.

Was the 2016 WHO classification also used for the diagnosis of patients presenting before the publication of this classification?

→Absolutely. All patients were re-diagnosed when the paper was written to ensure uniformity of diagnosis by WHO2016.

The authors do not need to add trade/brand names or give examples of HNB products. However, they must define what HNB is and how it differs from cigarette smoking.

→Thank you for pointed out.

The patient section was revised to clarify that the recruited participants were cigarette smokers with polycythemia. I hope that this makes it clear from the flow of the text that smoking means cigarette smoking, while HNB tobacco refers to heat-not-burn tobacco.

**Results**

Lines 138-147 are perhaps better suited for the method section. These are not senso stricto ‘results’.

→I'm sorry. For the sake of the flow of the article, I will just put it in the results section.

Line 196-198: Please revise the sentence for clarification. Do the authors mean to say “In patients who resumed cigarette consumption after smoking cessation, their Hct levels returned to the same level as at enrolment in this study?

- This statement is irrelevant if there is no data.

→I have the data. I added it to Manuscript.

**Discussion**

Line 228: “Therefore, smoking cessation is more urgent for those with polycythemia vera compared with those with smokers’ polycythemia.” This stands in contrast to what the authors are trying to say in the introduction lines 62-63. Why is it more urgent for those with PV when the authors discuss in the introduction that PV and secondary erythrocytosis have equivalent rates of thrombosis?

I apologize for the confusion. This is not contrast.

The incidence of thrombosis in patients with polycythemia vera (non-smoker PV) is the same as that in patients with smoker's polycythemia. However, it is well known that the incidence of arterial thrombosis in patients with polycythemia vera who smoke is 1.9 times higher than in patients who do not smoke. Thus, among patients with polycythemia vera, smokers have a higher risk of thrombosis than non-smokers.

To clarify this point, I have revised the sentence.

→The incidence of arterial thrombosis in patients with polycythemia vera who smoke is 1.9 times higher than in patients who are non-smokers [26, 27], and whose high Hct levels also increase the risk of thrombosis [28]. In this study, it was intriguing that in two polycythemia vera patients who have dropped out of smoking cessation programs, high Hct levels improved after switching to HNB tobacco without additional treatment.

Line 231: The results of this study ONLY SUGGESTS that HNB tobacco MAY be useful in decreasing the Hct and smoking-related symptoms in patients with **secondary polycythaemia**. No claims can be made with regards to PV and certainly not with regards to using HNB tobacco as “an emergency measure in patients with polycythemia vera…” to reduce the Hct. This claim is too bold with only 2 patients in the PV arm and no sufficient scientific evidence to make this statement. I ask that the authors revise this statement. The only evidence from this particulate study is that 2 patients with confirmed PV who switched to HNB tobacco had lower Hct levels after the switch.

→No other reports have described a decrease in Hct levels in patients with confirmed PV who switched to HNB cigarettes. Although the present study included two cases, this fact alone is important, so I have revised the sentence to remove any exaggerated claims.

Lines 232 – 239 are irrelevant to the study. The only qualification the authors have to make is that HNB, like normal cigarettes, contains harmful substances and the long-term effects have not yet been clarified.

The long-term effects are not described. Ten years have passed since HNB cigarettes were released, and new findings have emerged; therefore, the newer findings may differ from those of previous reports.

However, the following two points have been reported:

- The smoke of HNB cigarettes contains fewer harmful substances compared with paper cigarettes.

-Compared to smokers of paper cigarette, smokers of HNB cigarettes exhibit less DNA methylation of certain cancer-related genes.

The present report indicates that the health effects of HNB cigarettes remain unclear. We believe that the more information we provide our readers about HNB tobacco, the better, as a criterion for its application in clinical practice. Although this is often the subject of debate, there is no intention to mislead readers.

Line 242: Again. Although the results of this study are interesting, especially with regard to secondary polycythaemia, this study ONLY SUGGESTS that Hct MAY be reduced by HNB tobacco use compared to cigarette smoking in patients with secondary erythrocytosis. No conclusions can be drawn about PV or thrombosis risk. Further studies are needed to assess a reduction in the risk of thrombosis.

→I corrected this sentence to say only that it improved Hct.

→In this study, it was intriguing that in two polycythemia vera patients who have dropped out of smoking cessation programs, high Hct levels improved after switching to HNB tobacco without additional treatment.

I disagree with the authors that HNB tobacco “may become a therapeutic aid” (line 76) or is considered a “treatment option” (line 247). This could be misleading to many readers of this article. At best, HNB tobacco should be considered as an alternative to cessation of cigarette smoking when cessation of cigarette smoking is unsuccessful.

→I have revised this sentence as follows. However, I do not believe the original text is mistaken considering the trends in recent research reports.

Line 76: Herein, we report that switching to HNB tobacco improves polycythemia and subjective symptoms in smokers.

Line 247: Although complete smoking cessation is the best treatment, HNB tobacco may be considered as an alternative when cigarette smoking cessation is unsuccessful.

**Figure 1:**

- “Differential diagnosis not performed” as a reason for excluding 14 patients. What does this mean? The reason(s) for their exclusion is not clear.

**→**These are patients for whom consent was obtained, but for whom the attending physician had not performed sufficient tests for differential diagnosis. Some of the tests to check for obesity, hyperthyroidism, diabetes, or high Epo levels were not done. In addition, the medical interview did not mention whether the patient was experiencing mental stress.

- What is being referred to with “mental storess at work”? Does this refer to how psychological stress was assessed? How was this determined to sufficiently exclude the patient from the study?

**→**The patient was under considerable stress at work, and his polycythemia improved when he was transferred to another department. This occurred when multiple blood samples were collected before switching to HNB.
