# Supplementary material for: Introducing heat-not-burn tobacco improves hematocrit and cigarette smoking-related symptoms in patients with smokers’ polycythemia and polycythemia vera
Source: PLoS One. 2025 May 28;20(5):e0323437. doi: 10.1371/journal.pone.0323437 (PMC12118817; doi:10.1371/journal.pone.0323437)
Supplement: Attachment — Submitted filename: Response to Reviewers_comments_for_revised3.docx [file pone.0323437.s011.docx]

**Response to Reviewer**

We wish to express our appreciation to the Reviewer for his or her insightful comments, which have helped us significantly improve the paper.

Reviewer #1: Despite the study limitations, this manuscript is interesting and will be useful. I emphasize that the author should add limitations and recommendations
the results of this study suggest well that HNB tobacco may be so helpful in smoking-related symptoms in patients secondary polycythemia. But for smoker polycythemia vera patients, although the result in study is intriguing, it is also metaphorical. To reach the intended conclusion, the author would need to compare results between groups of smoker with polycythemia vera patient who are on treatment (who have fail to quit smoking and who switch to HNB tobacco), mentioning limitations here is demanding

Answer: It is corrected that by adding "However, due to the small sample size, further expanded studies are needed." to the "Discussion" section, we avoid misleading the readers and indicate that this is a topic for future research.

Reviewer #2: The article has been considerably improved since the first revision. At this stage, only minor revisions are necessary.

Minor revisions (line citation based on marked-up document):
Introduction
Line 56: The authors have changed the text here, so abbreviations must first be defined before they are used e.g. PV before using it for the first time and when it is defined, the authors do not need to write it in full (e.g. line 58). Please see the rest of the text for similar instances.

Answer: Thank you for pointing this out. We have corrected it as you pointed out.

Results:
Lines 186-189: This seems to be a mistake. Repetition of the previous lines.
Answer: Thank you for pointing this out. We have deleted the phrase.

Comments from previous review:

Reviewer comment:

The authors do not need to add trade/brand names or give examples of HNB products. However, they must define what HNB is and how it differs from cigarette smoking.

• The authors have not clearly defined HNB or explained how it differs from traditional cigarette smoke, assuming prior reader knowledge. Simply listing brand names is insufficient; a brief comparison should be included in my opinion. Important distinctions such as combustion in cigarettes (high temperature, smoke and by-products) versus heating in HNB (lower temperature, no combustion, aerosol production) would improve clarity. If the editor(s) feel that this level of detail is unnecessary, I am open to the authors not providing any additional response. Of note, the differences in CO content are addressed in the discussion.
Answer: We have reconsidered and added the following text to the “Introduction” section. We also removed the brand name.

“The characteristic of HNB tobacco is no combustion, lower temperature than cigarettes and do not produce smoke containing CO. On the other hand, metal aerosols are generated.”

• I still disagree that this needs to be in the results section (the first paragraph of the results section under “Patients"), but if the editor(s) are happy with the placement of the text on metholodology (describing the inclusion and exclusion of patients) in the results section, then I will overlook this response. In my opinion, adding this information to the methods section would improve "flow of the article”.

Answer: We have reconsidered and added it to the “Methods” section as your suggested.
