# Supplementary material for: Introducing heat-not-burn tobacco improves hematocrit and cigarette smoking-related symptoms in patients with smokers’ polycythemia and polycythemia vera
Source: PLoS One. 2025 May 28;20(5):e0323437. doi: 10.1371/journal.pone.0323437 (PMC12118817; doi:10.1371/journal.pone.0323437)
Supplement: Attachment — Submitted filename: Reviewer comments HNB vs smoking cessation.docx [file pone.0323437.s006.docx]

**Abstract**

- Objectives as a first heading should be changed to Introduction instead.
- Remove “on”.
- Methods section in abstract requires refinement.

**Introduction**

- A reference at the end of the sentence would suffice and prevent the flow of the sentence from being interrupted.
- Also consider HIF as a mechanism for absolute erythrocytosis (esp in patients with COPD)
  - However, the authors should mention and refer to reputable sources that EPO is low in smokers, in contrast to other causes of secondary polycythaemia where an elevated serum EPO level is to be expected.
- Grammar and sentence structures should be checked to ensure that the correct words and terms match the meaning/intention of the sentences. This seems to have been lost in the translation of the manuscript.

**Methods:**

- Please write genes and fusion genes such as “*BCR::ABL1*” in Italics instead of “BCR-ABL”
- FISH sensitivity to detect *BCR::ABL1* is very low; why was PCR not used and were there any clinicopathological features of CML in any of these patients?
- Define “SRL” before using it as an abbreviation.
- Provide more detail in the “Gene analysis” section.
  - Was a positive/mutant (heterozygous/homozygous), a negative/wild-type and a reagent blank control used during the *JAK2* mutation test?
  - Please state whether the PCR products (end-point analysis) were read on gel cards.
- Define what exactly is meant by ‘heat-not-burn’ (HNB) tobacco in the method section under “Treatment administration and assessment of outcome”.
- Was the serum EPO level recorded, if not, why not?
- Which programs/software were used for the statistical analysis and graphical representation of the data?
- How was the sputum volume objectively measured or is this also a subjective assumption?
- Hct is the only objective measure of improvement.
  - Was % blood oxygen saturation assessed for each of the patients at baseline and follow-up?
- The authors mention a mechanism of reduced plasma cell volume with cigarette smoking. Was systemic blood pressure affected by the switch to HNB or smoking cessation?
- Were baseline lung function tests performed?
- Is there a follow-up planned for incidence of thromboembolism in these patients?

**Results**

Table 1 – Total number is 12 but HNB is 8 and smoking cessation is 5 which equals 13. Was the one patient that had an unsuccessful smoking cessation, excluded from the data? I agree with the exclusion, but the authors need to state this exclusion and change the values in the table.

**Discussion**

“One possible mechanism is that absolute erythrocytosis which activates thrombin21 and increases platelet P secretin expression,22 which in turn increases the risk of thrombosis.” Did the authors mean “P selectin”?

“As the risk of secondary carcinogenesis related to cigarettes, cigarette smoking increases the incidence of polycythemia vera,26,27 as well as 16 types of cancer: lung, upper aerodigestive tract (oral cavity, nasal cavity, nasal sinus, pharynx, larynx), esophagus, stomach, pancreas, liver, kidney, lower urinary tract (renal pelvis and urinary bladder), and uterine cervix cancers, myelogenous leukemia, and myeloproliferative neoplasms. 28” This sentence seems incomplete.

Any number less than 10 can be written out in full, unless it is a measured value. E.g “…observed between the 2 groups before the change in smoking habits” should be written as two instead of 2.

A comment in the discussion section and in the summary should make it clear that there is no evidence that tobacco products that are not burnt are less harmful than cigarettes and that these products should be used with caution. These products should also not be endorsed by health professionals.

The methodology and results of this study are sound and contain very interesting findings, but the text needs a grammar/sentence reconstruction/spell check revision before this article is considered for publication.
