# Supplementary material for: Introducing heat-not-burn tobacco improves hematocrit and cigarette smoking-related symptoms in patients with smokers’ polycythemia and polycythemia vera
Source: PLoS One. 2025 May 28;20(5):e0323437. doi: 10.1371/journal.pone.0323437 (PMC12118817; doi:10.1371/journal.pone.0323437)
Supplement: Attachment — Submitted filename: Reviewer comments for revised manuscript EG.docx [file pone.0323437.s008.docx]

I have reviewed the revised manuscript and again suggest major revisions to the manuscript especially with regards to the claims on HNB tobacco use.

Could the authors of this study please provide a conflict-of-interest statement.

HUGO Gene Nomenclature Committee (HGNC) recommendations for the designation of gene fusions, recommends writing a fusion gene as ***BCR::ABL1****.*

- Bruford, E.A., Antonescu, C.R., Carroll, A.J. et al. HUGO Gene Nomenclature Committee (HGNC) recommendations for the designation of gene fusions. Leukemia 35, 3040–3043 (2021). <https://doi.org/10.1038/s41375-021-01436-6>
- Also see Nomenclature under the Submission guidelines for PLOS ONE (<https://journals.plos.org/plosone/s/submission-guidelines>)

The term ‘variant’ should be used instead of ‘mutation’ throughout the manuscript.

Subject to the requirements of PLOS ONE, it should be sufficient to place a reference at the end of the sentence and to improve the "flow" of the sentence.

**Abstract**

Line 35: write 2 as “two” instead.

**Introduction**

Line 52: mechanism or plural mechanisms? Change the grammar accordingly.

Line 58: Treatment for smokers’ polycythaemia?

Line 59: suggest using “detection or analysis or testing” rather than “measurement”

Line 60-61: Do the authors mean that the distinction/differentiation between a diagnosis of PV and secondary polycythaemia has become more accurate?

Line 59-63: This needs clarification. Do the authors mean that because we are now able to better distinguish between smokers’ (i.e secondary) polycythaemia and polycythaemia vera by using JAK2 variant analysis, that the results of the study in reference 10, in which JAK2 testing was performed, is more accurate than the 1993 report (case-control study), in determining which condition is more associated with thrombosis?

If this is the case, I suggest that the authors instead mention that the fact that JAK2 variants were not tested for is a limitation of the 1993 report and not the reason why the results of reference 10 suggest an equivalent thrombosis rate. Example: “In contrast to these earlier reports, where PV was not confirmed by testing for the JAK2 variant, a more recent study has shown that secondary polycythaemia and polycythaemia vera have comparable rates of thromboembolic events. This suggests that secondary polycythaemia also requires intervention to reduce the risk of thrombosis in this cohort.”

[The authors should also consider this article (Nadeem O, Gui J, Ornstein DL. Prevalence of venous thromboembolism in patients with secondary polycythemia. Clin Appl Thromb Hemost. 2013;19(4):363-366. doi:10.1177/1076029612460425) which states that “…secondary polycythemia alone may not be a significant risk factor for VTE but that VTE risk in this population may be related to known risk factors such as obesity.”]

Line 68: I suggest: “…increased the need for consultation with a hematologist.”

Line 75-77: The authors are making a bold statement about HNB tobacco as a ‘therapeutic aid’. Please rephrase this statement.

**Method**

Line 112-113: Add the abbreviation after polymerase chain reaction (PCR), which is used later in the paragraph, and move the AS abbreviation to allele-specific instead of putting it after 'polymerase'.

Please define FISH in the manuscript before using the abbreviation and provide sufficient methodological details in the manuscript for “FISH at SRL, Inc” and the PCR used for JAK2 testing.

Was the 2016 WHO classification also used for the diagnosis of patients presenting before the publication of this classification?

The authors do not need to add trade/brand names or give examples of HNB products. However, they must define what HNB is and how it differs from cigarette smoking.

**Results**

Lines 138-147 are perhaps better suited for the method section. These are not senso stricto ‘results’.

Line 196-198: Please revise the sentence for clarification. Do the authors mean to say “In patients who resumed cigarette consumption after smoking cessation, their Hct levels returned to the same level as at enrolment in this study?

- This statement is irrelevant if there is no data.

**Discussion**

Line 228: “Therefore, smoking cessation is more urgent for those with polycythemia vera compared with those with smokers’ polycythemia.” This stands in contrast to what the authors are trying to say in the introduction lines 62-63. Why is it more urgent for those with PV when the authors discuss in the introduction that PV and secondary erythrocytosis have equivalent rates of thrombosis?

Line 231: The results of this study ONLY SUGGESTS that HNB tobacco MAY be useful in decreasing the Hct and smoking-related symptoms in patients with **secondary polycythaemia**. No claims can be made with regards to PV and certainly not with regards to using HNB tobacco as “an emergency measure in patients with polycythemia vera…” to reduce the Hct. This claim is too bold with only 2 patients in the PV arm and no sufficient scientific evidence to make this statement. I ask that the authors revise this statement. The only evidence from this particulate study is that 2 patients with confirmed PV who switched to HNB tobacco had lower Hct levels after the switch.

Lines 232 – 239 are irrelevant to the study. The only qualification the authors have to make is that HNB, like normal cigarettes, contains harmful substances and the long-term effects have not yet been clarified.

Line 242: Again. Although the results of this study are interesting, especially with regard to secondary polycythaemia, this study ONLY SUGGESTS that Hct MAY be reduced by HNB tobacco use compared to cigarette smoking in patients with secondary erythrocytosis. No conclusions can be drawn about PV or thrombosis risk. Further studies are needed to assess a reduction in the risk of thrombosis.

I disagree with the authors that HNB tobacco “may become a therapeutic aid” (line 76) or is considered a “treatment option” (line 247). This could be misleading to many readers of this article. At best, HNB tobacco should be considered as an alternative to cessation of cigarette smoking when cessation of cigarette smoking is unsuccessful.

**Figure 1:**

- “Differential diagnosis not performed” as a reason for excluding 14 patients. What does this mean? The reason(s) for their exclusion is not clear.
- What is being referred to with “mental storess at work”? Does this refer to how psychological stress was assessed? How was this determined to sufficiently exclude the patient from the study?
